# Supplementary material for: Multidimensional mutual information methods for the analysis of covariation in multiple sequence alignments
Source: BMC Bioinformatics. 2014 May 22;15:157. doi: 10.1186/1471-2105-15-157 (PMC4046016; doi:10.1186/1471-2105-15-157)
Supplement: Additional file 1 — Derivation of 4-dimensional MI and averaged results for the 9 protein families studied. [file 1471-2105-15-157-S1.docx]

# Additional file 1

## Figure S1 - Detection of close contacts by covariation maps: averaged results for all protein families.


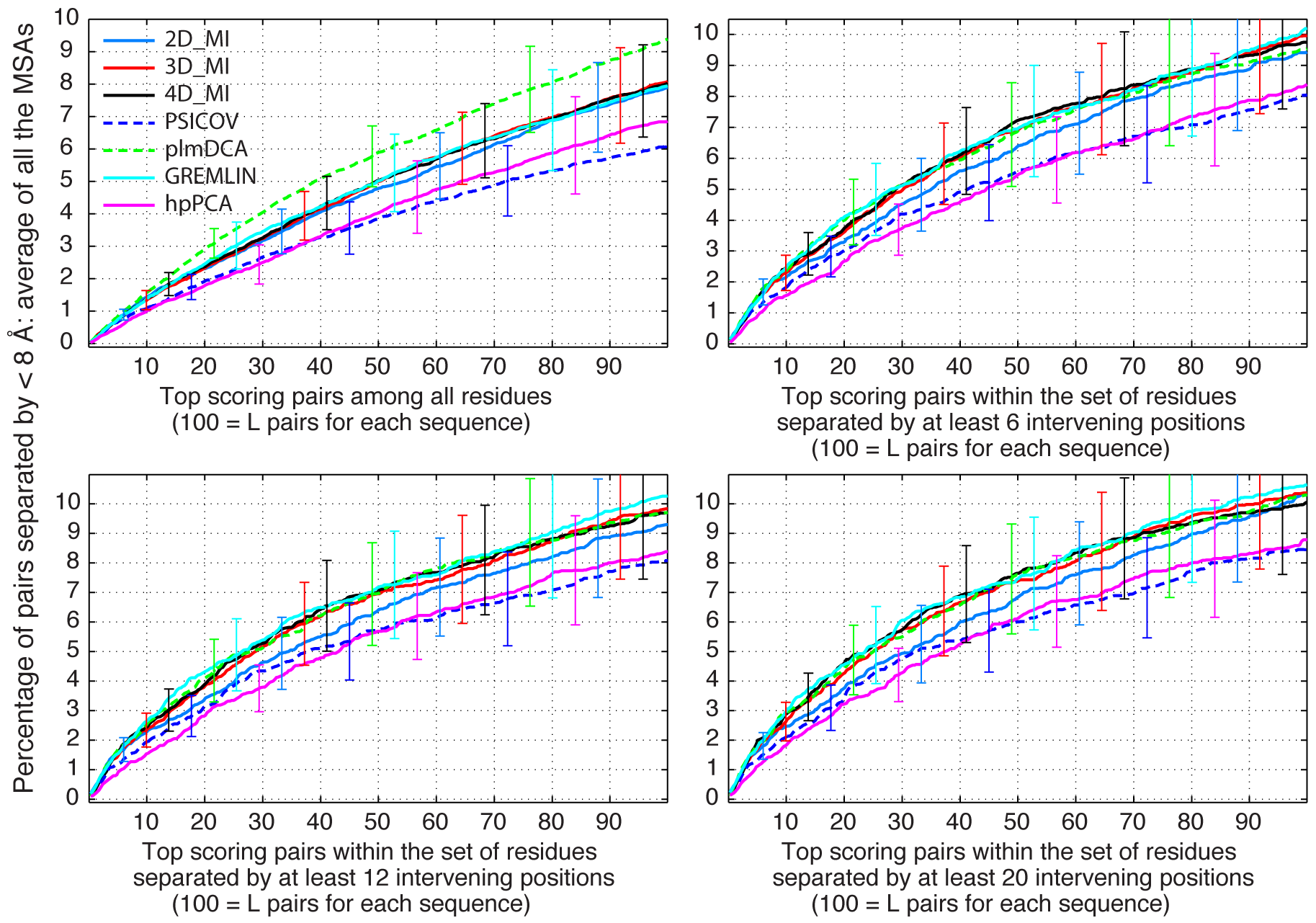


### Averaged results for the set of 9 protein families. Each panel shows what percentage of the residue pairs separated by less than 8 Å in the X-ray structure of each protein is present on average in the top covarying pairs identified by each method. The abscissa scale is normalized such that 100 corresponds to a number of pairs equal to the number of residues in each sequence. A. All protein pairs. B,C,D. Only pairs whose residues are separated by at least 6, 12, 20 intervening positions in sequence space.

**Figure S2 - Dependence of covariation scores on path length connectivity: average of all protein families.**

**
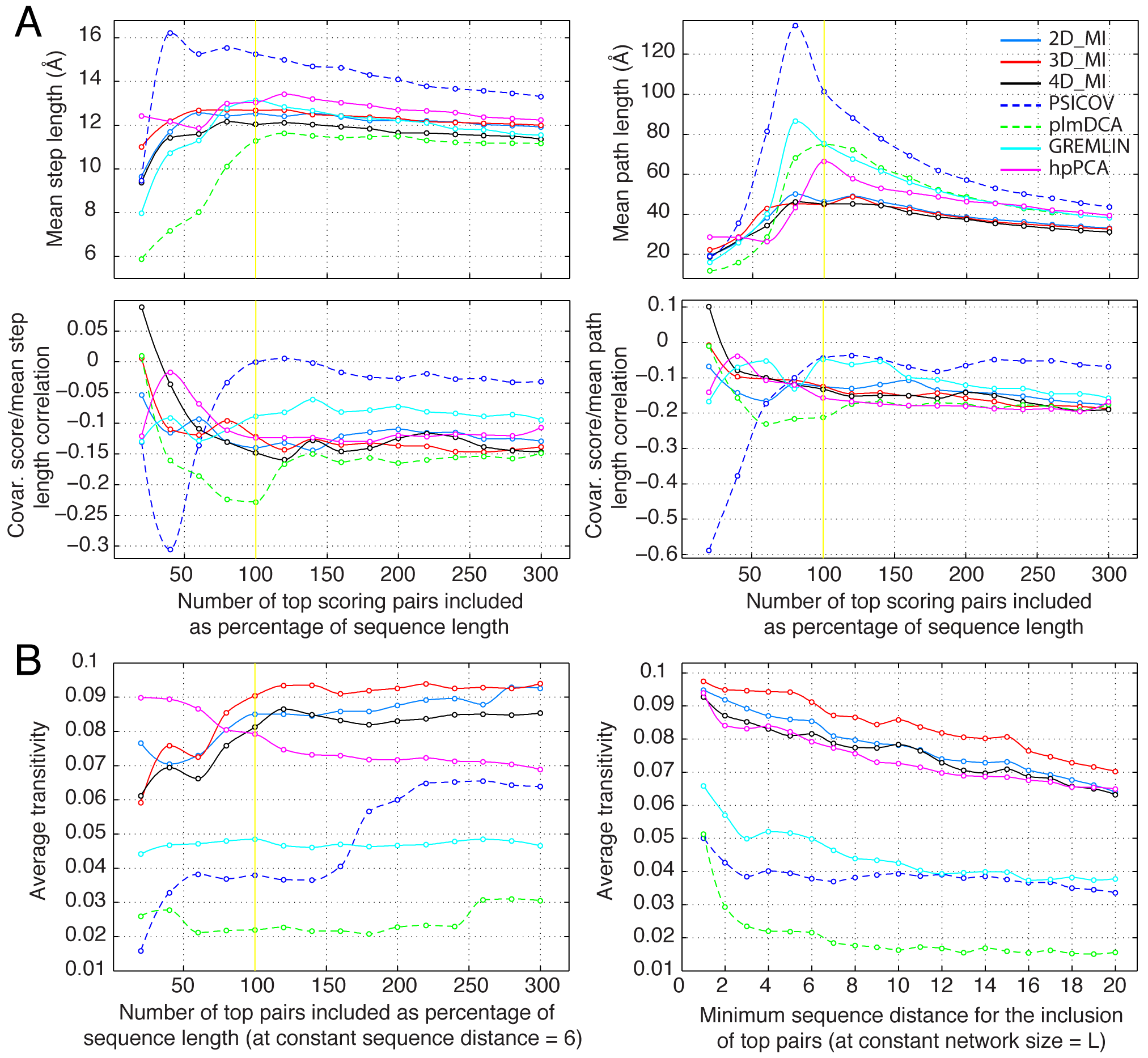
**

**A. Dependence of covariation scores on path length. Top left.** Mean length of the steps in each path connecting pairs within scoring threshold. The threshold is progressively moved to include a number of pairs equal to 3*L*. A vertical yellow line marks a number of pairs equal to *L*. **Top right.** Mean length of the path connecting pairs within scoring threshold. As more pairs are included in the analysis the probability of finding a shorter path increases and all traces converge to a smaller path length. **Bottom panels.** Correlation between the covariation score and the mean step length (**left panel**) or the total length (**righ panel**) of the path that connect the two members of each pairs through residues that belong to other pairs.

**B. Dependence of covariation scores on transitivity. Left panel.**  The size of the covariation graph is varied by including a progressively larger number of top scoring pairs at constant minimum sequence distance = 6. A vertical yellow line marks a number of pairs equal to *L*. **Right panel.** The minimum sequence distance is varied at constant graph size = *L*.

**Text S1 - Derivation of 4-dimensional MI.**

Mutual information I_X3,X4_(X1; X2) between X1 and X2, when the effect of two additional variables X3 and X4 on the transmission between them is removed, is obtained as:

I_X3,X4_(X1; X2 ) = ∑*p*_x3,x4_ I(X1; X2 | X3 = x3,X4 = x4) = I(X1; X2 | X3, X4) (6)

By the 'chain property' of multivariate MI we derive:

I(X1; X2 | X3, X4) = I(X1; X2 | X4) − I(X1; X2; X3 | X4)

= I(X1,X2) − I(X1;X2;X4) − I(X1;X2;X3) + I(X1;X2;X3;X4) (7)

where the 'interaction information' between the four variables is:

I(X1;X3;X2;X4) = [H(X1) + H(X2) + H(X3) + H(X4)] − [H(X1,X2) + H(X1,X3) + H(X1,X4) + H(X2, X3) + H(X2,X4) + H(X3,X4)] + [H(X1,X2,X3) + H(X1,X2,X4) + H(X1,X3,X4) + H(X2,X3,X4)] − H(X1,X2,X3,X4) (8)

Averaging over all values of X3 and X4 (two additional columns of the MSA), and recalling that all the values taken by X3 and X4 are the same with respect to X1 and X2 in a MSA, we finally obtain:

<I_X3,X4_(X1;X2)>_X3,X4≠X1,X2_ = I(X1;X2) − 2<I(X1;X2;X3)>_X3≠X1,X2_ + <I(X1;X2;X3;X4)>_X3,X4≠X1,X2_ (9)

Expanding (7) leads to a direct expression of I_X3,X4_(X1;X2) in terms of the entropies of the individual variables:

I_X3,X4_(X1;X2) = H(X1) + H(X2) − H(X1,X2)

− [H(X1) + H(X2) + H(X4 ) − H(X1, X2) − H(X1, X4)

− H(X2, X4) + H(X1, X2, X4)]

− [H(X1) + H(X2) + H(X3) − H(X1, X2) − H(X1, X3)

− H(X2, X3) + H(X1, X2, X3)]

+ [H(X1) + H(X2) + H(X3) + H(X4)] − [H(X1,X2) + H(X1,X3) + H(X1,X4) + H(X2, X3) + H(X2,X4) + H(X3,X4)] + [H(X1,X2,X3) + H(X1,X2,X4)

+ H(X1,X3,X4) + H(X2,X3,X4)] − H(X1,X2,X3,X4)

= ~~H(X1)~~ + ~~H(X2)~~ − ~~H(X1,X2)~~

−~~H(X1)~~ − ~~H(X2)~~ − ~~H(X4)~~ + ~~H(X1, X2)~~ + ~~H(X1, X4)~~

+ ~~H(X2, X4)~~ − ~~H(X1, X2, X4)~~

− ~~H(X1)~~ − ~~H(X2)~~ − ~~H(X3)~~ + ~~H(X1, X2)~~ + ~~H(X1, X3)~~

+ ~~H(X2, X3)~~ − ~~H(X1, X2, X3)~~

+ ~~H(X1)~~ + ~~H(X2)~~ + ~~H(X3)~~ + ~~H(X4)~~ − ~~H(X1,X2)~~ − ~~H(X1,X3)~~ − ~~H(X1,X4)~~

− ~~H(X2, X3)~~ − ~~H(X2,X4)~~ − H(X3,X4) + ~~H(X1,X2,X3)~~ + ~~H(X1,X2,X4)~~

+ H(X1,X3,X4) + H(X2,X3,X4) − H(X1,X2,X3,X4)

which simplifies to:

I_X3,X4_(X1;X2) = − H(X3,X4) + H(X1,X3,X4) + H(X2,X3,X4) − H(X1,X2,X3,X4) (10)
